# Comparing field-collected versus remotely-sensed variables to model malaria risk in the highlands of western Uganda

**DOI:** 10.1186/s12936-023-04628-w

**Published:** 2023-06-26

**Authors:** Brandon D. Hollingsworth, Hilary Sandborn, Emmanuel Baguma, Emmanuel Ayebare, Moses Ntaro, Edgar M. Mulogo, Ross M. Boyce

**Affiliations:** 1 Department of Entomology, Cornell University, Ithaca, NY 14850 USA; 2 Department of Geography, University of North Carolina at Chapel Hill, Chapel Hill, NC 27599 USA; 3 Department of Community Health, Faculty of Medicine, Mbarara University of Science & Technology, Mbarara, Uganda; 4 Institute for Global Health and Infectious Diseases, University of North Carolina at Chapel Hill, Chapel Hill, NC 27599 USA; 5 Department of Epidemiology, Gillings School of Global Public Health, University of North Carolina at Chapel Hill, Chapel Hill, NC 27599 USA; 6 Carolina Population Center, University of North Carolina at Chapel Hill, Chapel Hill, NC USA

**Keywords:** Malaria, Uganda, Micro-epidemiology

## Abstract

**Background:**

Malaria risk is not uniform across relatively small geographic areas, such as within a village. This heterogeneity in risk is associated with factors including demographic characteristics, individual behaviours, home construction, and environmental conditions, the importance of which varies by setting, making prediction difficult. This study attempted to compare the ability of statistical models to predict malaria risk at the household level using either (i) free easily-obtained remotely-sensed data or (ii) results from a resource-intensive household survey.

**Methods:**

The results of a household malaria survey conducted in 3 villages in western Uganda were combined with remotely-sensed environmental data to develop predictive models of two outcomes of interest (1) a positive ultrasensitive rapid diagnostic test (uRDT) and (2) inpatient admission for malaria within the last year. Generalized additive models were fit to each result using factors from the remotely-sensed data, the household survey, or a combination of both. Using a cross-validation approach, each model’s ability to predict malaria risk for out-of-sample households (OOS) and villages (OOV) was evaluated.

**Results:**

Models fit using only environmental variables provided a better fit and higher OOS predictive power for uRDT result (AIC = 362, AUC = 0.736) and inpatient admission (AIC = 623, AUC = 0.672) compared to models using household variables (uRDT AIC = 376, Admission AIC = 644, uRDT AUC = 0.667, Admission AUC = 0.653). Combining the datasets did not result in a better fit or higher OOS predictive power for uRDT results (AIC = 367, AUC = 0.671), but did for inpatient admission (AIC = 615, AUC = 0.683). Household factors performed best when predicting OOV uRDT results (AUC = 0.596) and inpatient admission (AUC = 0.553), but not much better than a random classifier.

**Conclusions:**

These results suggest that residual malaria risk is driven more by the external environment than home construction within the study area, possibly due to transmission regularly occurring outside of the home. Additionally, they suggest that when predicting malaria risk the benefit may not outweigh the high costs of attaining detailed information on household predictors. Instead, using remotely-sensed data provides an equally effective, cost-efficient alternative.

**Supplementary Information:**

The online version contains supplementary material available at 10.1186/s12936-023-04628-w.

## Background

*Plasmodium falciparum* malaria remains an important cause of global morbidity and mortality, accounting for an estimated 200 million annual cases and 600,000 deaths in the Africa alone [1]. While significant progress against malaria has been made, largely due to the widespread distribution and use of long-lasting insecticidal nets (LLINs), there is increasing evidence that progress has stalled in many of the highest burden settings [1]. Heterogeneity in bloodmeal-seeking behaviours (i.e., location and timing of feeding) may place an upper bound on the effectiveness of LLINs [2–4] and result in proportionally more bites occurring outdoors following LLIN deployment [5]. This has major implications for predicting residual malaria risk following LLIN deployment.

Therefore, an understanding of the factors beyond LLIN availability and use that are associated with malaria transmission remains necessary to target control measures effectively [6, 7]. Uganda has been a leader in the effort to achieve universal coverage of LLINs and is therefore an interesting setting to examine the factors associated with residual malaria risk [8]. The country conducted its first mass distribution campaign in 2013, [9] followed by similar campaigns every three years, including in 2017–18 and most recently in 2020–21 [10]. Remarkably, households reporting at least one LLIN increased from 16% in the 2006 Demographic and Health Survey (DHS) to more than 80% in the 2018 Malaria Indicator Survey (MIS), while over the same period the proportion of households with at least one LLIN for every two people increased from 5 to 54% [11]. Despite this progress, malaria transmission persists with more than 12 million cases reported in 2020 [1].

Malaria transmission intensity varies across Uganda, but individual and household risk may differ substantially even within a relatively small geographic area. Risk is impacted by numerous demographic, occupational, behavioural, and geographic factors occurring on different spatial and temporal scales which makes risk prediction, especially at fine-scale resolution, difficult [12]. Yet, understanding this fine scale spatial heterogeneity, which may be best explained by environmental conditions in the immediate peri-domestic space as well as household socio-economic factors, is critically important. How to most effectively identify and incorporate these variables into predictive models is not well defined.

Therefore, the goal of this study was to compare the ability of statistical models to predict malaria risk at the household level using either (i) remotely-sensed data or (ii) results from a household survey. These two methods of collection differ in both (1) the resources required to obtain the information and (2) the scale over which the predictors act. For example, information about home construction is costly to obtain and likely impacts risk within the home but not for neighbours. On the other hand, the presence of flooded areas is easily detected and may impact risk for a large area but is unlikely to explain differences in risk between neighbours. Given the level of detail, it was hypothesized that the inclusion of information collected in household surveys would result in higher predictive ability compared with only using remotely-sensed environmental data.

## Methods

### Setting

The Bugoye sub-county is located in the Kasese District of Western Uganda. With an area of approximately 55 km.^2^, this rural, highland area is comprised of 35 villages. The population of the sub-county is nearly 42,000, 17% of whom are children under 5 years of age [13]. The area is characterized by its varied geography, with deep river valleys and steep hillsides reaching elevations reaching 2500 m. The climate in western Uganda permits year-round malaria transmission with semi-annual peaks after the rainy seasons in May and November [14, 15] driven by a mixture of *Anopheles gambia*e, *Anopheles arabiensis*, and *Anopheles funestus*, among others [16]. The most recent MIS in the Tooro subnational region (2018–2019), which includes the sub-county, reported a PfPR of 7.3% although rates of 30% are reported in low-lying villages located along the river basins [11, 17].

### Household survey

The three participating villages were purposefully selected to achieve diversity in geography and malaria transmission intensity as determined by a previous survey of all 35 villages [17]. One village, Rwakingi 1A (PfPR_2-10_ 18.6%) was chosen because of its generally flat, flood-prone terrain adjacent to the Mubuku River. In contrast, Bunyangoni village (PfPR_2-10_ 10.5%), sits at the foothills of the Rwenzori mountains with a rapid increase in elevation from approximately 1200 m to 1600 m. Lastly, Kasanzi village (PfPR_2-10_ 31.7%) was chosen because a spillway from a nearby hydroelectric plant runs through the village, which was hypothesized to possibly be a man-made driver of malaria risk, given the intermittent nature of water flow through the canal.

**Fig. 1:**
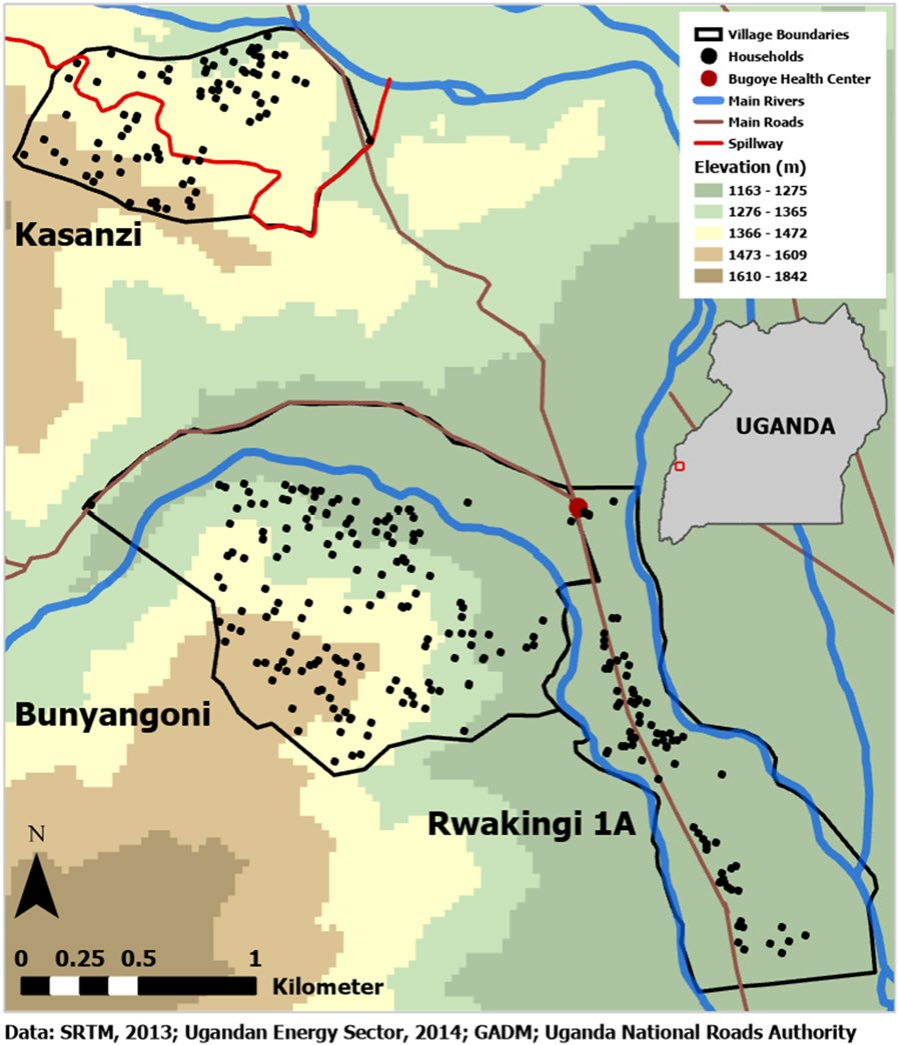
Elevation map of the Kasanzi, Bunyangoni, and Rwakingi 1A villages in the Bugoye sub-county displaying the location of surveyed households

In collaboration with local community health workers, all households in each village (Fig. 1) were visited between November 3rd, 2020 and November 24th, 2020, a time that aligned with the traditional second rainy period of the year. At each household, field staff provided detailed information about the objectives, eligibility criteria, methods, and risks/benefits of the study to an adult caregiver. Individuals agreeing to participate were asked to provide written consent. Demographic information was collected from all participating household members. Additionally, adult caregivers were asked to provide written consent for a finger-prick blood draw from all eligible children aged 2–12 years, while children ≥ 8 years also provided written assent. Participants received a household identifier card to track subsequent malaria infections and a small incentive (e.g. $2–3 or small in-kind items) to offset the opportunity cost of completing the survey. If no adult was present at the time of the visit, the survey team recorded the location and moved to the next household. Three attempts were made to revisit any households and all eligible individuals residing in the household were included.

At each participating household, field staff documented the household location using a GPS-equipped device and administered a questionnaire modified from the most recent DHS household questionnaire [11]. This questionnaire included information on household construction, water sources, toilet location, the ownership of various animals and durable goods, and the use of LLINs and indoor residual spraying. Wealth components were calculated for each household using principal component analysis of survey results, similar to the DHS wealth index, [18] but retaining the first two principal components. While several options were given for water sources, no household with children reported a water source other than “piped water” or “surface water”, so it was reduced to a binary variable. Staff measured axillary temperature and drew approximately 250 µl of capillary blood from all children 2–12 years of age via finger-prick or heel stick. Approximately 50 µl of blood drawn was used for an Alere Malaria Ag ultra-sensitive rapid diagnostic test (uRDT) (Abbott Laboratories, USA) [19]. The uRDT is a qualitative test for the detection of histidine-rich protein II (HRP-II) antigen of *P. falciparum* in human whole blood. All uRDT were obtained directly from the manufacturer, used prior to the date of expiry, and performed in accordance with the manufacturer’s instructions. Children with fever (axillary temperature ≥ 38° Celsius) and a positive uRDT received weight-based treatment with artemether lumefantrine in accordance with local treatment guidelines [20]. All information was recorded and uploaded to a secure electronic database (i.e., REDCap) using smart phones with cellular internet connectivity [21].

### Environmental data

Elevation, slope, and flow direction were derived from the Shuttle Radar Topography Mission 30 m Digital Elevation Model [22] using ArcGIS Pro (v. 2.7.0) [23]. (Additional file 1) Slope is a measurement of steepness of the ground surface, calculated as ${\text{Slope}}={\mathrm{tan}}^{-1}\left(\frac{\Delta {\text{Elevation}}}{\text{Distance}}\right)$. Flow direction is the direction in which water would flow out of the cell, corresponding roughly to the downhill direction, given in compass direction with east designated as 0. To account for the cyclical nature of compass direction, e.g., both 0 and 360 correspond to east, sine and cosine transforms were used. Normalized difference vegetation index (NDVI) was derived from U.S. Geological Survey Landsat 8 30 m imagery from December 12, 2020 [24]. To account for human and vector movement, environmental variables, except distance to the nearest river, were averaged across buffer regions with radii of 0 m, 100 m, 250 m, 500 m, 1000 m, 1500 m, and 2000 m around each participant’s residence. Only the buffer sizes that produced the lowest Akaike information criteria (AIC) values during model fitting were used for subsequent statistical analysis. Additionally, the Euclidean distances in meters were calculated from each household point location to the nearest river [25], the Level III Bugoye Health Centre, and the Kasanzi spillway. Distance to the level III health centre, the only public facility in the sub-county where inpatient care is available, is expected to affect the likelihood of care seeking but not risk of malaria exposure, and thus not an individual’s uRDT result. For this reason, distance to the health centre was included in models of inpatient admission only and excluded when predicting the spatial distribution of malaria risk.

### Data analysis

To compare the relative importance of the two predictive datasets, models were fit using three different sets of explanatory variables to two outcomes of interest; (i) uRDT positivity and (ii) inpatient admission for malaria (i.e., severe malaria) within the last year. The explanatory variables were divided into environmental or household variables, with the exception of latitude and longitude, which were included in all models, and distance to level III health facility, which was included in all models of inpatient admission (Table 1). A third set combined both sets of variables. Generalized additive models (GAM) with a logit-link function were fit using the mgcv package [26] in R v. 4.2.0 [27]. Splines were used within the model for latitude, longitude, elevation, NDVI, slope, distance to river, distance to level III health facility, and the first two principal components of wealth indicators. In addition, a tensor product smooth was included for the latitude by longitude interaction, and the flow direction sine and cosine interaction. Households were included as a random effect smooth to account for correlation in observations within a household. To determine the effect of distance to the Kasanzi spillway, distance to spill way was included with the environmental data and models were refit to individuals residing in Kasanzi. A basis dimension (k) of 3 was used to minimize overfitting in all models. All p-values are the result of Chi-squared tests, using either prop.test or anova functions in R.

**Table 1:** Explanatory variables included in the models

Variable	Description	Measurement or unit
All		
Latitude	Coordinate specifying the north–south position of a point on Earth’s surface	Decimal degrees
Longitude	Coordinate specifying the east–west position of a point on Earth’s surface	Decimal degrees
Distance to level III health facility (Inpatient admission only)	Euclidean distance from the household location to the Level III inpatient health facility	Meters
Environmental		
Village	Village of the household location	Kasanzi, Bunyangoni, or Rwakingi 1A
Elevation	Distance above sea level	Meters
Slope	Steepness of the ground surface;${\mathrm{tan}}^{-1}\left(\frac{\Delta {\text{Elevation}}}{\text{Distance}}\right)$	Degrees
Flow direction	Direction of flow out of each cell in a surface raster	Compass direction with 0 corresponding to east
NDVI	Difference between near-infrared (which vegetation reflects) and red light (which vegetation absorbs)	Range from -1 (bare ground or water) to + 1 (green vegetation)
Distance to nearest river	Euclidean distance from the household location to the nearest river	Meters
Household		
Age group	The age group of the child	1–5, 5–12, or 12–18 years old
Sex	The sex of the child	Male or female
Net	If the child slept under bed net the previous night	Yes or no
Door	If there is a door in the main room used for sleeping that leads outside	Yes or no
Window	If there is a window in the main room used for sleeping	Yes or no
Window screen	If there is a window, whether it completely closes or has screening or not	Yes or no
Eaves	If there are eaves (space between the roof and wall) in the main room used for sleeping	Yes or no
Eaves screen	If there are eaves, whether they have screening or not	Yes or no
Toilet location	Where the toilet is located	In own dwelling, in own yard or plot, or elsewhere
Water source	The main source of drinking water for household members	Piped water or surface water
Wealth 1	1^st^ Principal component of Household Survey	
Wealth 2	2^nd^ Principal component of household survey	
Inpatient admission^1^	If individual was admitted to an inpatient health facility for malaria within the last year	Yes or no
uRDT result^2^	Result of the URSDT administered during the survey	Positive or negative

^1^Inpatient admission was only used in predicting uRDT risk

^2^uRDT result was only used when predicting hospitalization risk

All models were initially fit to the full dataset with model selection determined by AIC. Models were compared across different buffer region radii and datasets and diagnostic plots were visually inspected for violations of model assumptions. To evaluate the out-of-sample (OOS) predictive ability of each model, cross-validation was performed using a random train-test split approach with an 80:20 split for 50 iterations. OOS predictions are made by training a model on a subset of data, then predicting the remaining data, providing an approximation of how the model performs when predicting novel data. The data were randomly split at the household level, with all models fit to the same training data. Finally, the out-of-village (OOV) predictive ability was determined by excluding each village in turn, fitting the models, and evaluating the model’s predictive ability within the excluded village. Predictive ability was compared based on the area under the curve (AUC) of the receiver-operator curve (ROC) when predicting the test dataset at each iteration [28]. These curves were calculated using the ROCR package [29].

### Ethical considerations

Ethical approval of the study was provided by the institutional review boards of the University of North Carolina at Chapel Hill (19-1094), the Mbarara University of Science and Technology (06/03-19), and the Uganda National Council for Science and Technology (HS 2628).

## Results

### Household survey

Results of the household survey are summarized by village in Table 2. The median age of individuals surveyed was 18 years (IQR: (6, 35)), and 62.3% were female. Demographic characteristics did not differ significantly between the villages. Reported LLIN usage for the previous night was high (> 90%) across all villages, likely reflecting the effect of an ongoing mass distribution campaign in 2020. The proportion of houses with screens on windows (p = 0.011,) and eaves (p = 0.008) was significantly different between villages, as was the proportion of houses with water piped into the house (p < 0.001). Further, the household survey found the highest rates of parasitemia amongst 2 to 12-year-olds in Kasanzi (21.5%), followed by Rwakingi 1A (15.9%), and Bunyangoni (4.8%). In contrast, children in Rwakingi 1A were the most likely to report having been admitted for malaria within the last year (42.8%), followed by Bunyangoni (32.6%) and Kasanzi (22.6%). While there are significant numbers of observations around household variables missing, they were exclusively from houses where no children resided and did not impact our results.

**Table 2:** Demographics and key descriptors of the three villages making up the study area

	Bunyangoni	Kasanzi	Rwakingi 1A
Environmental			
Elevation (m) (mean, IQR)	1325.09 (1246, 1395)	1419.13 (1383, 1448)	1201.62 (1188, 1214)
NDVI (mean, IQR)	0.389 (0.365, 0.415)	0.384 (0.361, 0.411)	0.367 (0.352, 0.406)
Population			
Census population	1299	507	774
Households (n)	186	106	133
Individuals Surveyed (n)	793	428	407
Age (median, IQR)	22 (6, 34)	18 (7, 35)	20 (6, 35)
Female (n, %)	394 (51.6%)	211 (51.5%)	175 (50.1%)
*Missing Sex (n)*	*30*	*18*	57
Children (n, %)	395 (51.4%)	206 (50.1%)	170 (48.6%)
* Missing Age (n)*	*24*	*17*	*57*
12–18 (n, %)	25 (3.3%)	21 (5.1%)	10 (2.9%)
5–12 (n, %)	240 (31.3%)	121 (29.4%)	98 (28.0%)
< 5 (n, %)	130 (16.9%)	64 (15.5%)	62 (17.7%)
% Sleeping Under LLIN	94.1%	92.7%	94.3%
Home construction			
Windows (n, %)	129 (70.9%)	75 (76.5%)	89 (90.8%)
* Missing (n)*	*4*	*8*	*46*
With screens (n, %)	86 (66.7%)	36 (48%)	61 (77.2%)
Eaves	80 (43.7%)	36 (36%)	24 (27.9%)
*Missing (n)*	*3*	*6*	*47*
With screens (n, %)	16 (20%)	0 (0%)	2 (8.3%)
Water piped to house (n, %)	82 (44.1%)	87 (82.1%)	87 (98.8%)
*Missing (n)*	*0*	*0*	*82*
Malaria outcomes			
uRDT + (n, %)	15 (4.8%)	37 (21.5%)	19 (16.3%)
* Total (n)*	*311*	*172*	*116*
Admitted w/in last year (n, %)	122 (32.5%)	43 (22.6%)	62 (42.8%)
* Total (n)*	*375*	*190*	*133*

*IQR* interquartile range, *LLIN* long-lasting insecticidal net, *NDVI* Normalized difference vegetation index, *uRDT* ultrasensitive rapid diagnostic test

Number of missing entries given in italics

### Predictive modeling

**Fig. 2:**
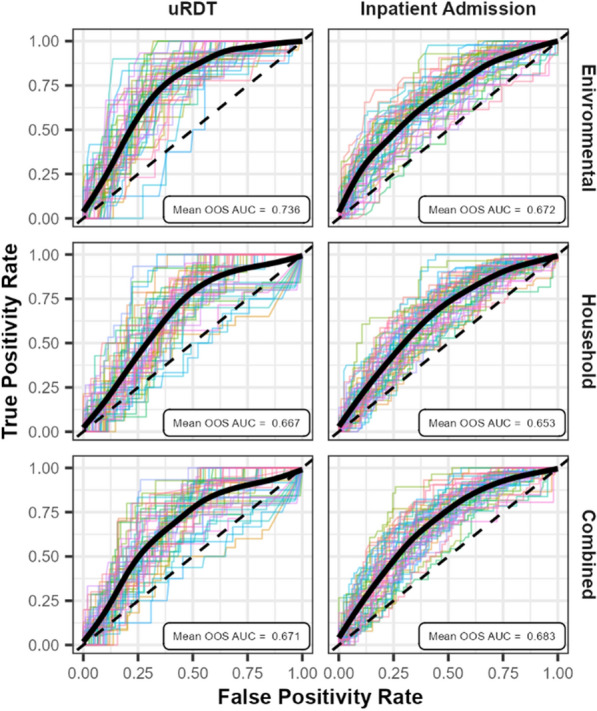
Receiver operating characteristic (ROC) curves for model fits. Result are shown for models predicting uRDT result (left column) and Inpatient Admission within the last year (right column) based on the environmental (top row), household (center row) and combined (bottom row) datasets. ROC curves for out-of-sample (OOS) predictions from 50 test-training splits are given (colored lines), along with the mean ROC curve (black line), and the mean OOS area under the curve (AUC). Diagonal dashed line shows the results of a random classifier. The environmental dataset best predicts OOS uRDT test results (mean OOS AUC = 0.736), while the combined dataset best predicts inpatient admission (mean OOS AUC = 0.683)

Buffer sizes of 250 m and 1500 m produced the best fit based on AIC for uRDT result and inpatient admissions, respectively, and were used in subsequent analysis (Additional file 1: Table S1). Choice of buffer size did not have a large impact on the model’s predictive power (Additional file 1: Table S1). The environmental dataset provided a significantly better fit to uRDT results (AIC = 362) and higher OOS predictive power (AUC = 0.736) compared to the household (AIC = 376, AUC = 0.667) and combined (AIC = 367, AUC = 0.671) datasets. For inpatient admissions, the combined model provided the best fit (AIC = 615) and OOS prediction (AUC = 0.683) compared to the environmental (AIC = 624, AUC = 0.672) and household (AIC = 644, AUC = 0.653) datasets. Figure 2 shows the ROC curves for each model. For OOV malaria risk, no dataset performed significantly better than a random classifier that naïvely assigns a state (e.g., uRDT +) to an individual based solely on an expected likelihood an individual is in that state (e.g., observed prevalence) (Additional file 1: Fig. S1).

**Fig. 3:**
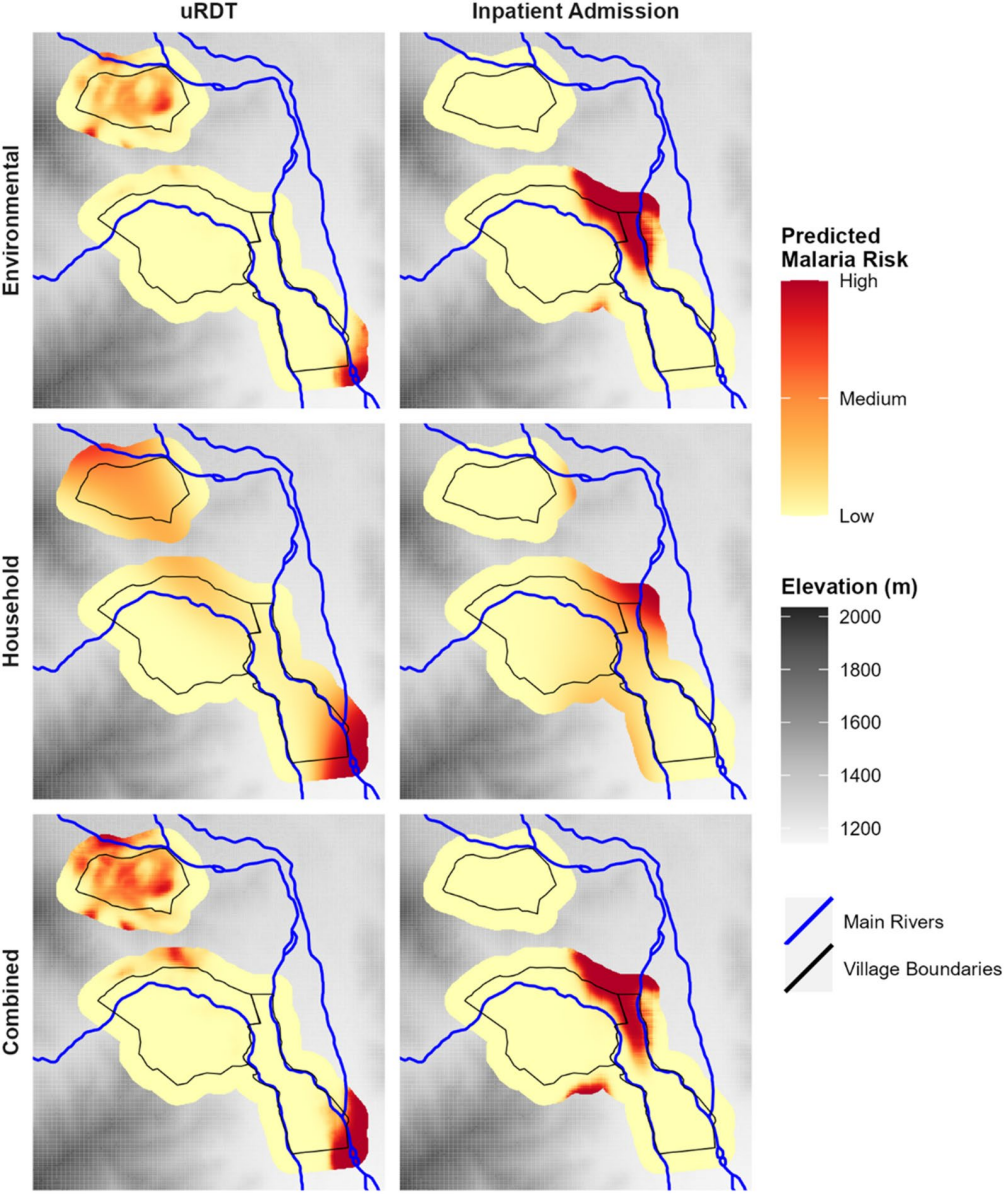
Environmental risk predicted by model fits. This is the risk assigned to environmental variables after accounting for any individual (e.g. age and sex) or household (e.g. home construction) level variables. Results are shown for models predicting uRDT result (left column) and Inpatient Admission within the last year (right column) based on the environmental (top row), household (center row) and combined (bottom row) datasets. Predictions are standardized across figures to allow comparison of areas of high and low risk. All three models predict that southeast Rwakingi 1A has the highest risk of positive uRDT results, followed with areas of Kasanzi. Results for risk of inpatient admission are more varied, but all models agree that the highest risk area is in Northern Rwakingi 1A and western Bunyangoni

Figure 3 shows the relative risk predicted by each model. All models predicted the highest risk of malaria infection (uRDT result) in southeastern Rwakingi 1A, a lowland region between two rivers that is prone to flooding. Predicted risk decreases from east to west through Rwakingi 1A and Bunyangoni as the landscape changes from lowlands along rivers to more mountainous areas with higher elevations, with the lowest risk predicted along the steep hillsides on the western slope of Bunyangoni. Similarly, all three models predict moderate to high risk in areas of central Kasanzi, an area that contains a manmade, concrete culvert used to divert excess water from a local hydroelectric plant. In contrast to the environmental and combined models, the household model predicts large areas of intermediate risk through Rwakingi 1A and northwestern Bunyangoni. This is likely a result of the household model smoothing across low-risk regions that fall between high-risk regions, since environmental factors are not included.

In contrast to the predicted risk of a positive uRDT result, the highest risk of inpatient admission for malaria is predicted in north Rwakingi 1A and northeastern Bunyangoni. This is likely due to the location of the Level III Bugoye Health Centre which is situated in that region on the border between Rwakingi 1A and Bunyangoni. A relatively low risk of inpatient admission was seen in Kasanzi, much of Bunyangoni, and south Rwakingi 1A. In contrast to the others, the household model predicts a quicker shift from high to moderate risk as you move away from northern Rwakingi 1A and northeastern Bunyangoni.

### Risk factors

The best fitting model for uRDT result, using the environmental dataset with a 250 m buffer, found that the house’s latitude (p = 0.026), flow direction (cosine) (p = 0.014), and flow direction (interaction) (p = 0.003) were all significant predictors of uRDT result. The relationships between these variables and uRDT result were found to be nonlinear. All estimated smooth response curves are shown in Additional file 1: Fig. S2. Within Kasanzi, the model found no evidence of an effect of distance from the spillway on uRDT results (p = 0.382). When combined with information from the household survey, all of these variables remained significant except for flow direction (cosine). The model using the combined dataset also found the latitude by longitude interaction (p = 0.049) and the flow direction (sine) (p = 0.035) to be significant predictors of malaria risk.

The best fitting model for predicting inpatient admission within the last year, the combined dataset with a 1500 m buffer, found that longitude (positive, p = 0.037), the latitude by longitude interaction (p = 0.041), slope (nonlinear, p = 0.024), flow direction (sine) (nonlinear, p = 0.008), and flow direction (interaction) (nonlinear, p = 0.029) significantly impacted outcomes. All estimated smooth response curves are shown in Additional file 1: Fig. S7. In addition, distance from the spillway was found to significantly impact outcomes within Kasanzi (Environmental Dataset: aRR = 1.04, p = 0.021, Combined Dataset: aRR = 1.07, p = 0.042). See Additional file 1: Tables S2–S3 and Additional file 1: Fig. S2-S7 for full results.

## Discussion

Using the results of a household malaria survey performed across three villages of differing terrain and malaria transmission in rural Uganda, the predictive ability of models for malaria risk were compared. The findings show that the environmental dataset outperforms the household dataset at predicting OOS malaria risk based on both uRDT result (mean AUC of 0.736 compared to 0.667) and inpatient admissions (mean AUC of 0.672 compared to 0.653). While this is not a large difference, the substantially higher cost of collecting the household dataset would heavily favor using the environmental dataset. In addition, while the inclusion of the household dataset with the environmental dataset (i.e., combined dataset) improved models’ ability to predict OOS inpatient admissions (mean AUC of 0.683 compared to 0.672), it actually decreased the ability to predict OOS uRDT results (mean AUC of 0.671). Importantly, no model outperformed a random classifier when predicting OOV risk (Additional file 1), highlighting the difficulty of extrapolating results to new regions, even in close proximity.

The datasets used here differed not just in the variables they contained, but in the costs associated with obtaining them. The environmental dataset contains variables that would be expected to predict the presence of vector habitat, such as standing water. This dataset is easily obtained from publicly available online tools (e.g., USGS EarthExplorer) and would be expected to best predict malaria risk if transmission primarily occurred outside the home, since it does not account for physical barriers (e.g., window screening, LLINs) limiting vector access to the individual inside the home. On the other hand, the household dataset is much more logistically difficult to obtain, requiring a detailed survey of households, and would be expected to best predict malaria risk if transmission primarily occurred inside the home (e.g., while individuals slept). In reality, malaria risk is expected to depend on a complex interaction between these variables, with their relative importance being location dependent. Therefore, it is important for policy-makers to understand the circumstances within their region, which requires at least a preliminary examination of all possible risk factors. Risk mapping is a valuable tool for malaria control, as it can identify high risk areas and guide surveillance, prevention and treatment activities, resource allocation [30].

The low impact of several household variables is partly due to the lack of variation between individuals tested. For example, of 608 children surveyed, 568 (93.4%) reported sleeping under a LLIN the previous night, while 567 (93.3%) lived in households with toilets on the property. While we did not measure entomological indices, one possible explanation for the low predictive power of household variables compared to environmental variables is that the high proportion of children sleeping under bed nets could result in a shift in where malaria transmission occurs, from within the house to outside, [2–5] lessening the ability of household variables to predict residual malaria risk. However, it has also been suggested that sufficient biting still occurs late at night within households with LLINs for transmission to occur [4, 5]. The high prevalence of LLIN during this study was almost certainly the result of a national LLIN mass distribution campaign in 2020–21 [40] and was significantly higher than observed in the region in January-March 2020, when coverage was found to be 64.7% [17]. In addition, utilization of protective measures within the household (e.g., LLIN and installation of screening) may reflect both actual risk and the perceived risk of the homeowner. Homeowners may install protective measures in response to either a perceived high malaria risk (e.g., living near the spillway) or an actual risk (e.g., seeing mosquitoes in their homes). Previous work has also found household variables to have counter-intuitive relationships with malaria risk in the presence of LLINs, [31] including a decreased risk of malaria associated with windows tied to cooler indoor temperatures and improved LLIN compliance. This association would become stronger if transmission occurs outside the household, where they are no longer protective.

The models found that slope and flow direction were significant predictors of both measures of malaria risk. Slope steepness and flow direction affect water accumulation, necessary for larval development, which has been previously shown to correlate with higher malaria risk [32–34]. In addition, larval habitat is known to be more common in locations closer to streams and rivers, [32] and proximity to water has been shown to influence malaria risk, both within Uganda [35, 36] and in other regions [37, 38], even after accounting for household construction [39]. Elevation has long been established as a predictor of malaria risk with risk decreasing at higher elevations [33, 40, 41] while this work finds no association of elevation with malaria risk, previous work in the region showing that low elevation villages have higher prevalence of infection [17, 42] and lower levels of multiplicity of infection [42] for malaria than high elevation villages, measures of malaria transmission intensity. Finally, it is well-established that distance to a health facility is a determinant of healthcare utilization in rural settings [43] resulting in individuals delaying or refusing to seek care, [44] self-medicating, [45] or seeking care outside the formal healthcare system [43]. While this was seen when using the environmental dataset, distance to the nearest level 3 health facility was not significant when household variables were included.

Several others have attempted to predict malaria risk using environmental and/or individual- and household-level variables across a number of settings [33, 46–49]. These models typically had similar levels of predictive power (AUC = 0.7–0.9). Despite this, there are key differences in the data included to produce these models when compared to the models in this study. Several studies similarly use remotely-sensed data to predict risk based on environmental variables, [12, 33, 46–49] but few combine this data with individual- and household-level information [46, 47]. For those that do use individual- and household-level data, few include house construction information [47]. Another key difference is that others have relied on aggregated malaria prevalence data [46], while our analyses used individual household-level malaria prevalence information. Thus, this study offers a unique set of variables for predicting risk. Additionally, few studies have compared the predictive ability of three subsets of environmental and individual- and household-level variables, which this study has done.

While this study provides a unique dataset with which to compare the predictive ability of several factors, there are several important limitations. First, the dataset represents a single household survey conducted in November 2020. This excludes the possibility of examining the effect of seasonality or short-term weather conditions. Second, a single NDVI estimate, derived from December 2020 data, was used for fitting the environmental models. NDVI varies over the year, driven by a bi-annual rainy season. This variation was not captured in the analysis. Third, this study uses uRDT test results and previous inpatient admission as outcomes. Given the persistence of HRP2, it is possible that infection could have occurred anytime in the 6 weeks prior to the uRDT test results [50]. Similarly, inpatient admission was assessed over the previous year. Thus, the risk factors present at the time of the study may not be representative of those present at the time the infection occurred. Finally, travel history was not collected as part of the household survey. Varying sizes of buffer regions around the households were included to account for areas individuals may visit, but it is not possible to adjust for individual-level variation in movement without a travel history.

## Conclusion

Accurate fine-scale prediction of malaria risk is essential, especially in regions where malaria persists despite high LLIN uptake. Many of these regions have limited resources that need to be proactively targeted towards areas of the greatest need. There is a growing body of work looking at the determinants of malaria risk at a household level, but building accurate models still proves difficult. Further developing these models not only requires technical advancements in modelling, e.g. machine learning, but an understanding of the scales, implications, and costs of different predictive datasets. To this end, the use of easily obtainable remotely-sensed environmental data has been compared to a dataset collected as part of a highly detailed household survey when predicting two indicators of malaria risk. It was found that environmental data were able to better predict OOS uRDT positivity and inpatient admission across three villages in Uganda and that the addition of household-level data provided marginal, if any, benefit. This has important implications for developing predictive models in the current environment as it suggests that the use of remotely-sensed data may be sufficient and that the added benefit of household surveys may not justify their costs. However, in areas with low LLIN coverage, or with limited environmental variation, household surveys are likely still necessary to understand variation in malaria risk.

## Supplementary Information

**Additional file 1: Table S1**: Akaike Information Criteria for each dataset. Values for models used in analysis are in bold. **Table S2**: Estimated relative risk and p-values for fixed effects in the best fit models for uRDT using a 250m buffer and inpatient admission using a 1500m buffer. For villages, age groups, and toilet location, values represent a comparison against the reference groups; Bunyangoni, 0-5 years, and not on property, respectively. For window and eave screens, this is risk relative to an unscreened window or eave. **Table S3** P-values and estimated degrees of freedom for smoothed terms and random effects in the best fit models for uRDT using a 250m buffer and inpatient admission using a 1500m buffer. **Figure S1**: Receiver operating characteristic curves for model fits. Result are shown for models predicting uRDT result and Inpatient Admission within the last year based on the environmental, household and combined datasets. ROC curves for out-of-village predictions from 3 test-training splits are given, along with the mean ROC curve, and the mean OOV area under the curve. Diagonal dashed line shows the results of a random classifier. The household dataset best predicts OOV uRDT test results and inpatient admission. **Figure S2**: Estimated smoothed fits for uRDT results using the environmental dataset. **Figure S3**: Estimated smoothed fits for uRDT results using the household dataset. **Figure S4**: Estimated smoothed fits for uRDT results using the combined dataset. **Figure S5**: Estimated smoothed fits for inpatient admission using the environmental dataset. **Figure S6**: Estimated smoothed fits for inpatient admission using the household dataset. **Figure S7**: Estimated smoothed fits for inpatient admission using the combined dataset. **Figure S8**: Diagnostic plots for model fits of uRDT results using the environmental dataset. **Figure S9**: Diagnostic plots for model fits of uRDT results using the household dataset. **Figure S10**: Diagnostic plots for model fits of uRDT results using the combined dataset. **Figure S11**: Diagnostic plots for model fits of inpatient admission results using the environmental dataset. **Figure S12**: Diagnostic plots for model fits of inpatient admission using the household dataset. **Figure S13**: Diagnostic plots for model fits of inpatient admission using the combined dataset.

## Data Availability

Deidentified individual data that supports the results will be shared beginning 9 to 36 months following publication provided the investigator who proposes to use the data has approval from an Institutional Review Board (IRB), Independent Ethics Committee (IEC), or Research Ethics Board (REB), as applicable, and executes a data use/sharing agreement with UNC.
