# Supplementary material for: Comparing field-collected versus remotely-sensed variables to model malaria risk in the highlands of western Uganda
Source: Malar J. 2023 Jun 26;22:197. doi: 10.1186/s12936-023-04628-w (PMC10294526; doi:10.1186/s12936-023-04628-w)
Supplement: Supplementary file 1 — Additional file 1: Table S1: Akaike Information Criteria for each dataset. Values for models used in analysis are in bold. Table S2: Estimated relative risk and p-values for fixed effects in the best fit models for uRDT using a 250m buffer and inpatient admission using a 1500m buffer. For villages, age groups, and toilet location, values represent a comparison against the reference groups; Bunyangoni, 0-5 years, and not on property, respectively. For window and eave screens, this is risk relative to an unscreened window or eave. Table S3 P-values and estimated degrees of freedom for smoothed terms and random effects in the best fit models for uRDT using a 250m buffer and inpatient admission using a 1500m buffer. Figure S1: Receiver operating characteristic curves for model fits. Result are shown for models predicting uRDT result and Inpatient Admission within the last year based on the environmental, household and combined datasets. ROC curves for out-of-village predictions from 3 test-training splits are given, along with the mean ROC curve, and the mean OOV area under the curve. Diagonal dashed line shows the results of a random classifier. The household dataset best predicts OOV uRDT test results and inpatient admission. Figure S2: Estimated smoothed fits for uRDT results using the environmental dataset. Figure S3: Estimated smoothed fits for uRDT results using the household dataset. Figure S4: Estimated smoothed fits for uRDT results using the combined dataset. Figure S5: Estimated smoothed fits for inpatient admission using the environmental dataset. Figure S6: Estimated smoothed fits for inpatient admission using the household dataset. Figure S7: Estimated smoothed fits for inpatient admission using the combined dataset. Figure S8: Diagnostic plots for model fits of uRDT results using the environmental dataset. Figure S9: Diagnostic plots for model fits of uRDT results using the household dataset. Figure S10: Diagnostic plots for model fits of uRDT results usi [file 12936_2023_4628_MOESM1_ESM.docx]

**Additional file**

**Additional file 1: Table S1** Akaike Information Criteria (AIC) for each dataset. Values for models used in analysis are in **bold**.

|  |  | **uRDT** | **Inpatient Admission** |
| --- | --- | --- | --- |
| **Data Set** | **Buffer Region** | **AIC** | **AIC** |
| **Environmental** | 0m | 363.54 | 659.37 |
|  | 100m | 387.98 | 645.76 |
|  | 250m | **361.56** | 663.17 |
|  | 500m | 375.69 | 646.92 |
|  | 1000m | 372.18 | 634.33 |
|  | 1500m | 373.61 | **623.70** |
|  | 2000m | 375.89 | 631.21 |
| **Household** | ------- | **376.14** | **644.08** |
| **Combined** | 0m | 362.05 | 631.19 |
|  | 100m | 376.43 | 627.27 |
|  | 250m | **366.77** | 635.70 |
|  | 500m | 376.80 | 626.31 |
|  | 1000m | 373.46 | 620.14 |
|  | 1500m | 375.23 | **615.10** |
|  | 2000m | 376.11 | 617.70 |

**Additional file 1: Table S2** Estimated relative risk and p-values for fixed effects in the best fit models for uRDT using a 250m buffer and inpatient admission using a 1500m buffer. For villages, age groups, and toilet location, values represent a comparison against the reference groups; Bunyangoni, 0-5 years, and not on property, respectively. For window and eave screens, this is risk relative to an unscreened window or eave.

|  | **uRDT** | | | **Inpatient Admission** | | |
| --- | --- | --- | --- | --- | --- | --- |
|  | Environmental | Household | Combined | Environmental | Household | Combined |
| Kasanzi | 0.0166  (0.226) | 0.150  (0.370) | 0.00796  (0.275) | 5.20  (0.660) | 7.22  (0.464) | (0.729) |
| Rwakingi 1A | 616  (0.098) | 1.01  (0.996) | 39.2  (0.314) | 0.0128  (0.076) | 0.564  (0.763) | (0.097) |
| 5-12 years |  | 1.39  (0.196) | 1.46  (0.166) |  | 1.13  (0.580) | 1.14  (0.570) |
| Male |  | 1.27  (0.455) | 1.16  (0.659) |  | 1.31  (0.371) | 1.40  (0.305) |
| Net Use |  | 0.781  (0.696) | 0.559  (0.414) |  | 2.10  (0.268) | 2.17  (0.283) |
| Window |  | 0.391  (0.105) | 0.260  (0.037) |  | 1.44  (0.608) | 1.98  (0.412) |
| Window Screen |  | 1.81  (0.230) | 1.93  (0.222) |  | 3.11  (0.047) | 3.26  (0.082) |
| Eaves |  | 0.883  (0.763) | 0.993  (0.988) |  | 0.997  (0.965) | 1.09  (0.893) |
| Eave Screen |  | 0.884  (0.922) | 0.552  (0.718) |  | 4.96  (0.116) | 3.83  (0.260) |
| Door |  | 0.648  (0.290) | 1.02  (0.962) |  | 0.369  (0.044) | .375  (0.093) |
| Toilet in dwelling |  | 0.0253  (0.029) | 0.0342  (0.070) |  | 1.72  (0.771) | 5.57  (0.437) |
| Toilet in yard |  | 0.0736  (0.040) | 0.0625  (0.046) |  | 0.664  (0.798) | 2.19  (0.668) |
| Water not piped in |  | 0.370  (0.144) | 0.585  (0.507) |  | 2.43  (0.225) | 1.70  (0.555) |
| Inpatient Admission |  | 1.49  (0.275) | 1.78  (0.141) |  |  |  |
| uRDT+ |  |  |  |  | 2.03  (0.175) | 2.32  (0.140) |

**Additional file 1:** Table S3 P-values and estimated degrees of freedom (edf) for smoothed terms and random effects in the best fit models for uRDT using a 250m buffer and inpatient admission using a 1500m buffer.

|  | **uRDT** | | | **Inpatient Admission** | | |
| --- | --- | --- | --- | --- | --- | --- |
|  | Environmental | Household | Combined | Environmental | Household | Combined |
| Household (random effect) | 0.007  --- | <0.001  --- | 0.007  --- | <0.001  --- | <0.001  --- | <0.001  --- |
| Latitude | 0.026  (2.00) | <0.001  (2.00) | 0.015  (2.00) | 0.028  (1.00) | 0.229  (1.00) | 0.095  (1.00) |
| Longitude | 0.089  (1.93) | 0.025  (1.99) | 0.074  (2.00) | 0.009  (1.00) | 0.089  (1.00) | 0.037  (1.00) |
| Latitude:  Longitude | 0.063  (2.00) | 0.004  (1.94) | 0.049  (1.94) | 0.003  (4.00) | 0.315  (4.00) | 0.041  (4.00) |
| Elevation | 0.205  (1.00) |  | 0.365  (1.00) | 0.102  (1.00) |  | 0.079  (1.00) |
| Distance to a river | 0.176  (1.00) |  | 0.230  (1.00) | 0.386  (2.00) |  | 0.149  (1.00) |
| NDVI | 0.490  (1.8) |  | 0.654  (1.00) | 0.326  (2.00) |  | 0.253  (1.00) |
| Slope | 0.112  (2.00) |  | 0.166  (2.00) | 0.020  (2.00) |  | 0.024  (2.00) |
| Flow Direction (sine) | 0.109  (1.00) |  | 0.035  (1.00) | <0.001  (2.00) |  | 0.008  (2.00) |
| Flow Direction (cosine) | 0.014  (2.00) |  | 0.065  (2.00) | 0.089  (1.00) |  | 0.072  (1.00) |
| Flow Direction  (interaction) | 0.003  (2.00) |  | 0.006  (2.00) | 0.003  (4.00) |  | 0.029  (4.00) |
| Distance to nearest level 3 health facility |  |  |  | 0.004  (2.00) | 0.332  (1.00) | 0.149  (1.00) |
| Wealth Component 1 |  | 0.361  (1.27) | 0.589  (1.65) |  | 0.779  (1.99) | 0.497  (2.00) |
| Wealth Component 2 |  | 0.321  (1.00) | 0.805  (1.00) |  | 0.238  (2.00) | 0.475  (2.00) |

**OOV Results**

**
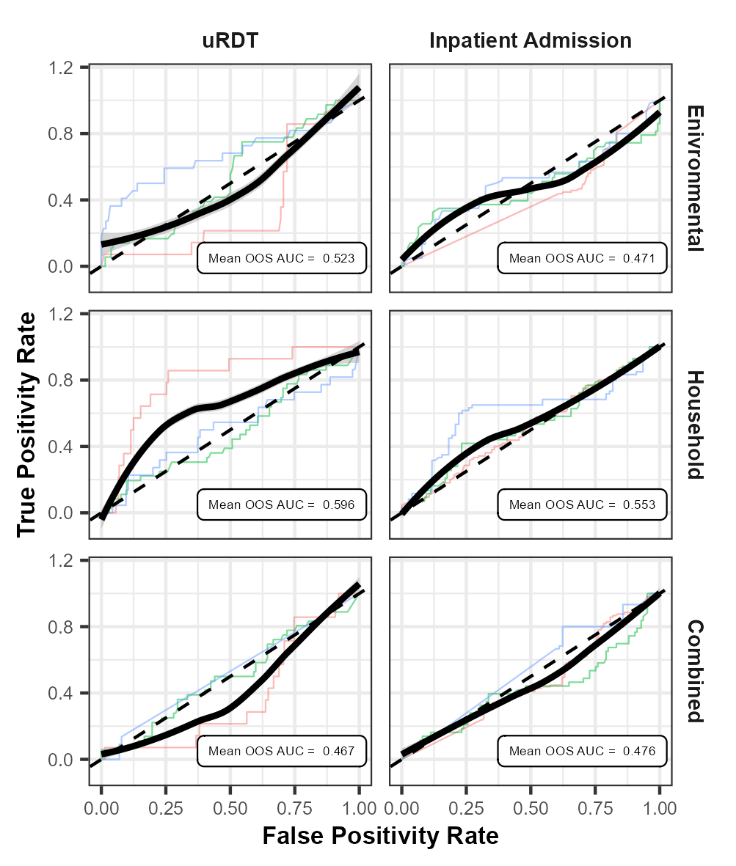
**

**Additional file 1: Figure S1:** Receiver operating characteristic (ROC) curves for model fits. Result are shown for models predicting uRDT result (left column) and Inpatient Admission within the last year (right column) based on the environmental (top row), household (center row) and combined (bottom row) datasets. ROC curves for out-of-village (OOV) predictions from 3 test-training splits are given (colored lines), along with the mean ROC curve (black line), and the mean OOV area under the curve (AUC). Diagonal dashed line shows the results of a random classifier. The household dataset best predicts OOV uRDT test results (mean OOV AUC = 0.596) and inpatient admission (mean OOV AUC = 0.553).

**Estimated smooths**


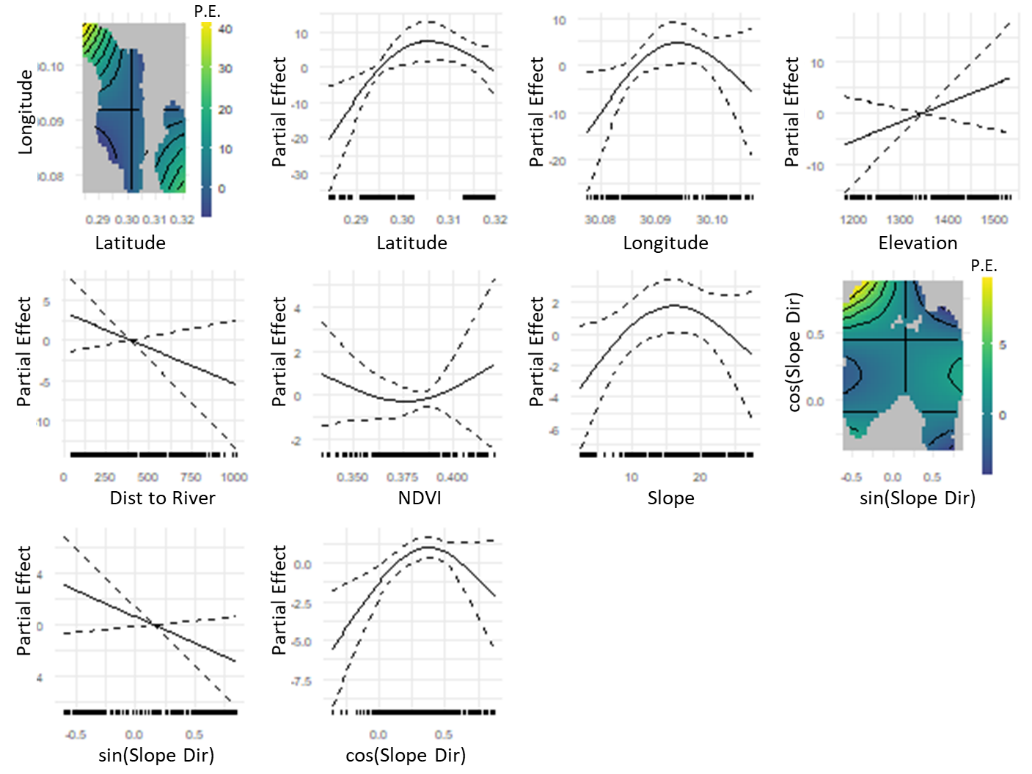


**Additional file 1: Figure S2:** Estimated smoothed fits for uRDT results using the environmental dataset.


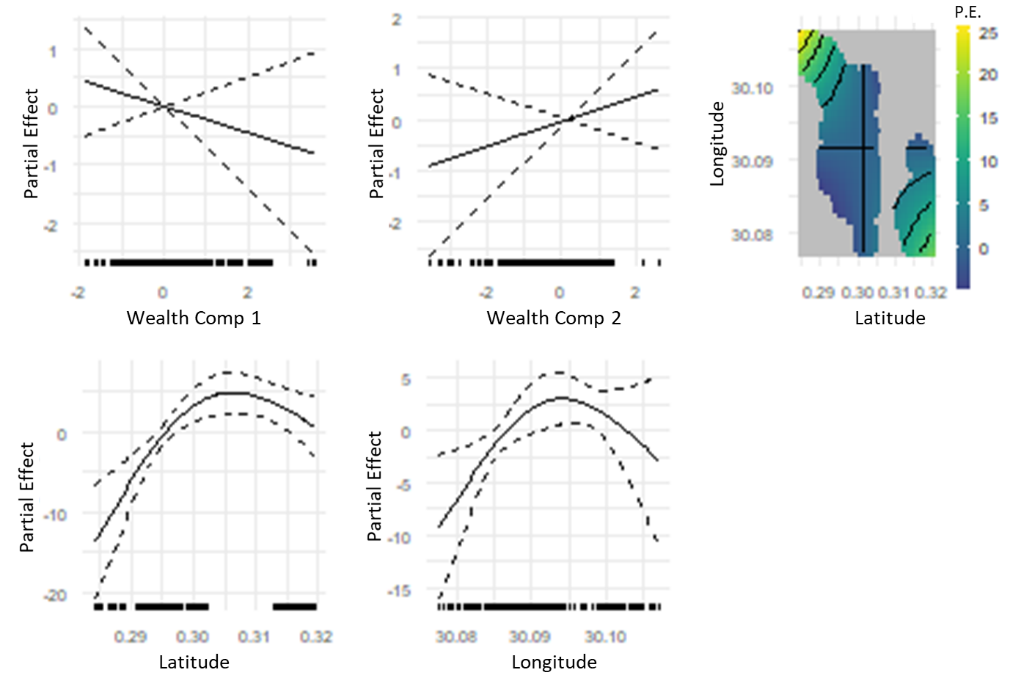


**Additional file 1: Figure S3:** Estimated smoothed fits for uRDT results using the household dataset.


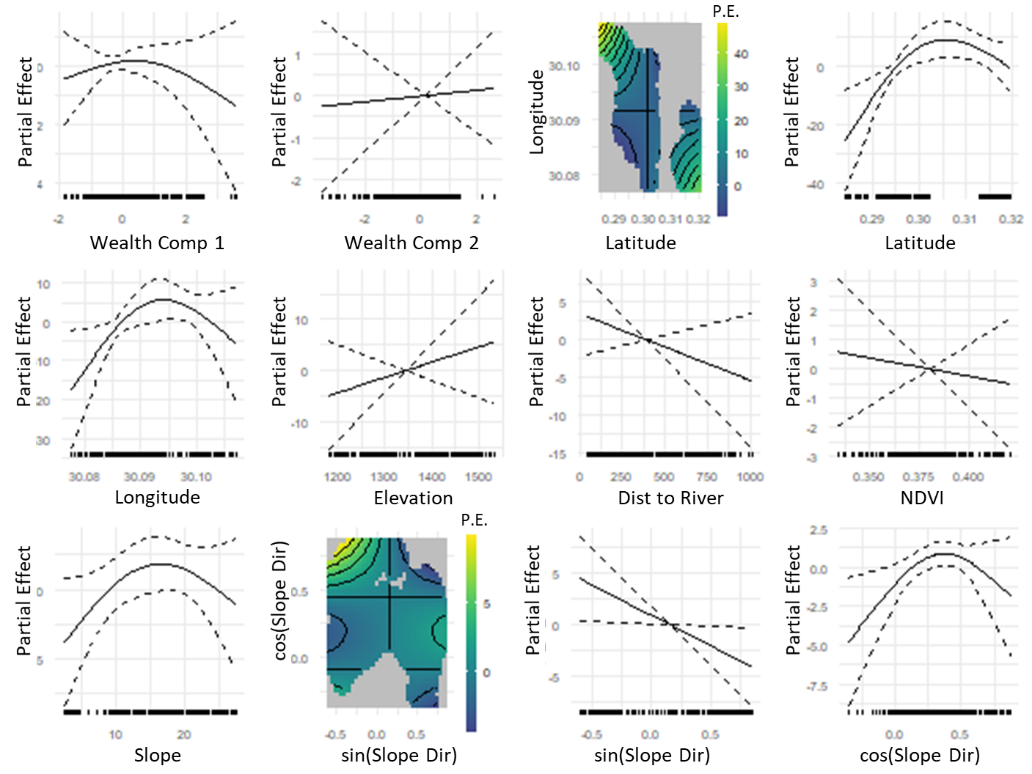


**Additional file 1: Figure S4:** Estimated smoothed fits for uRDT results using the combined dataset.


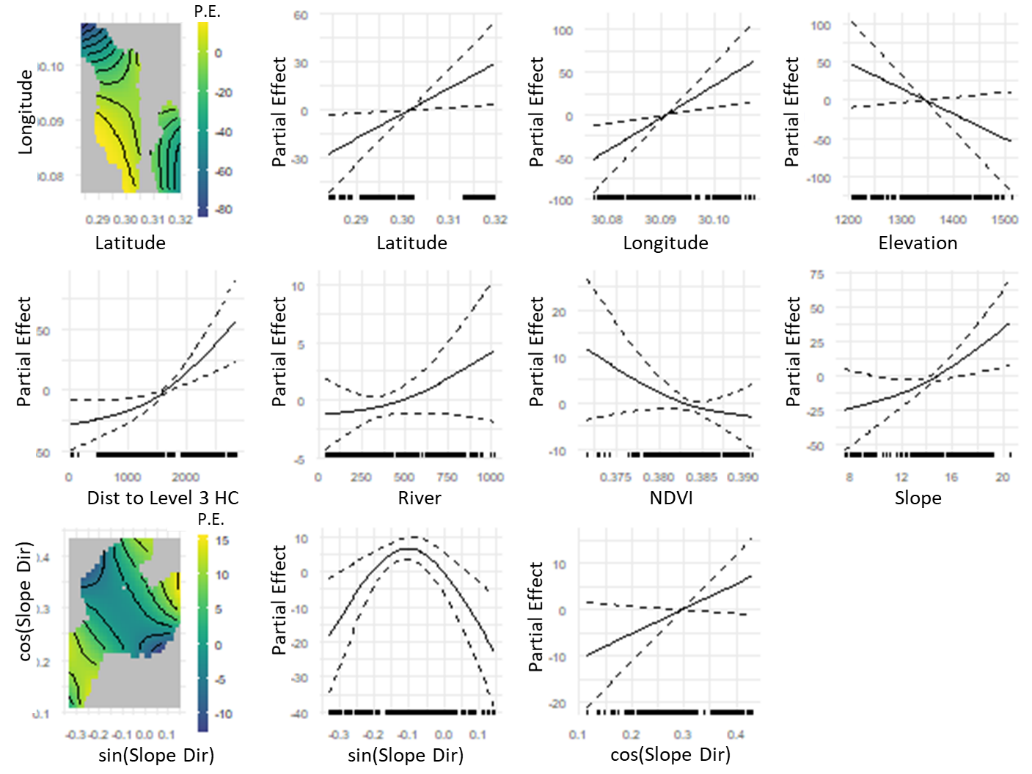


**Additional file 1: Figure S5:** Estimated smoothed fits for inpatient admission using the environmental dataset.


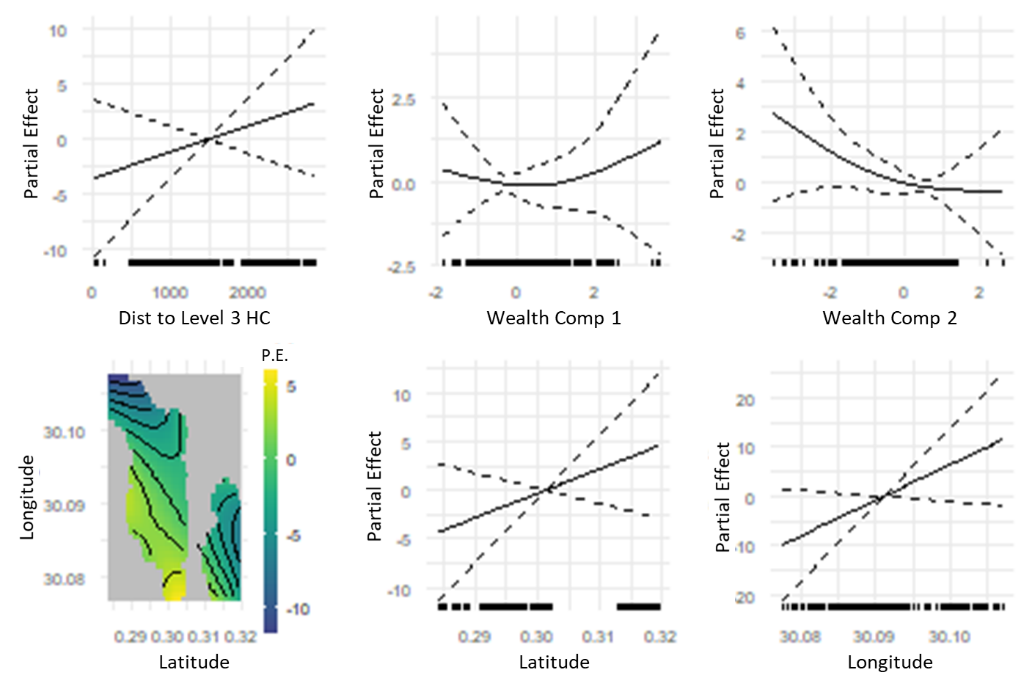


**Additional file 1: Figure S6:** Estimated smoothed fits for inpatient admission using the household dataset.


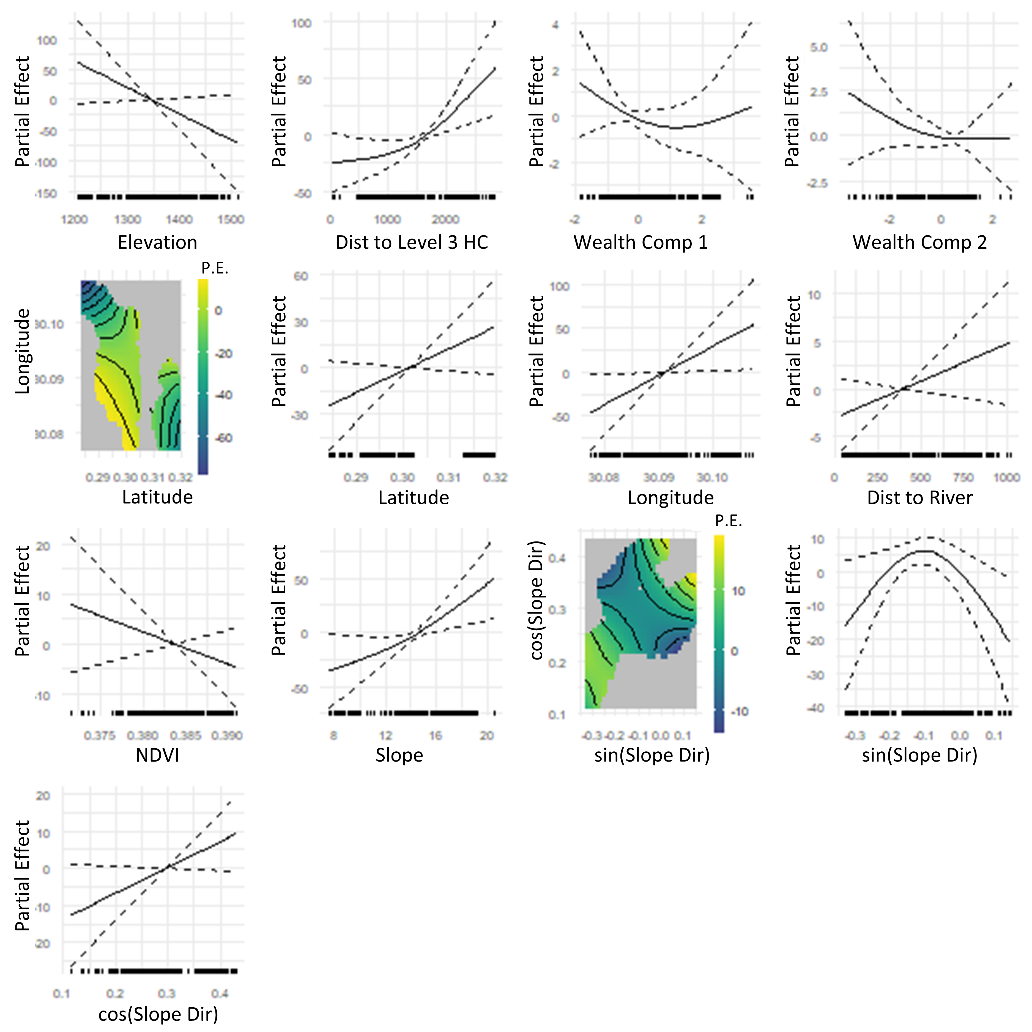


**Additional file 1: Figure S7:** Estimated smoothed fits for inpatient admission using the combined dataset.

Model Diagnostics:


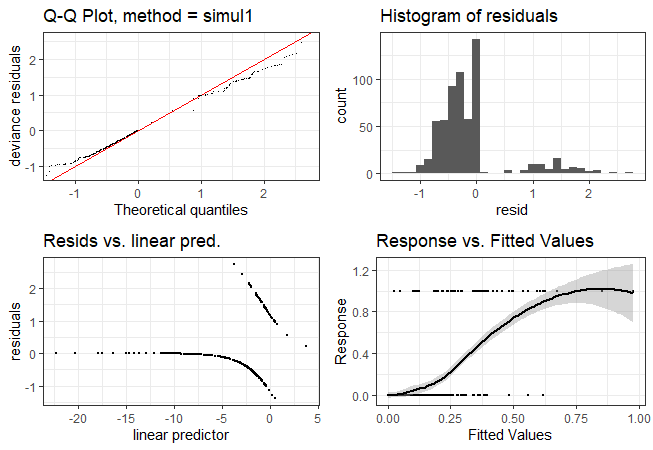


**Additional file 1: Figure S8:** Diagnostic plots for model fits of uRDT results using the environmental dataset.


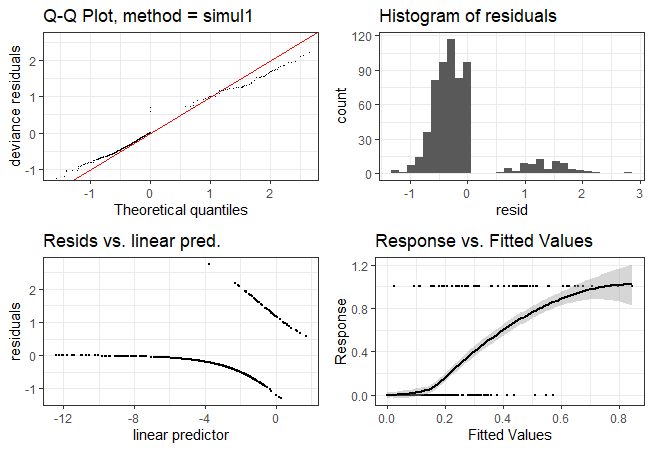


**Additional file 1: Figure S9:** Diagnostic plots for model fits of uRDT results using the household dataset.


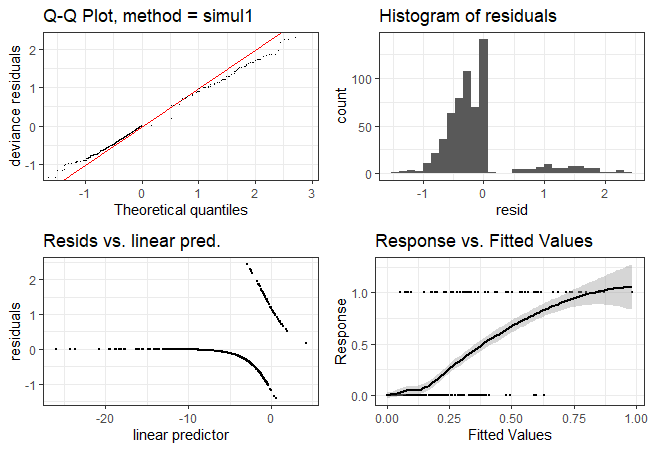


**Additional file 1: Figure S10:** Diagnostic plots for model fits of uRDT results using the combined dataset


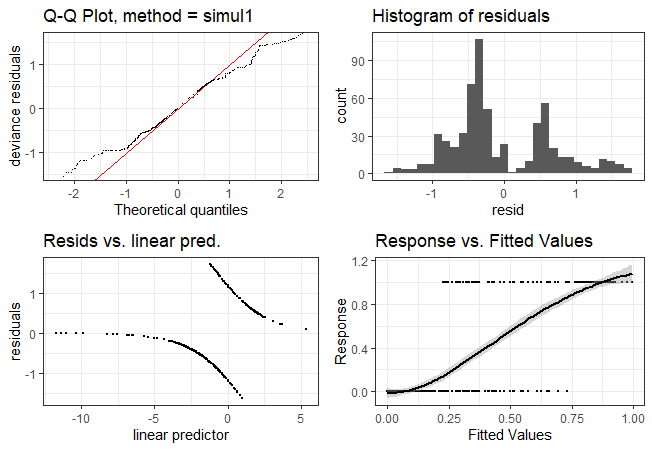


**Additional file 1: Figure S11:** Diagnostic plots for model fits of inpatient admission results using the environmental dataset


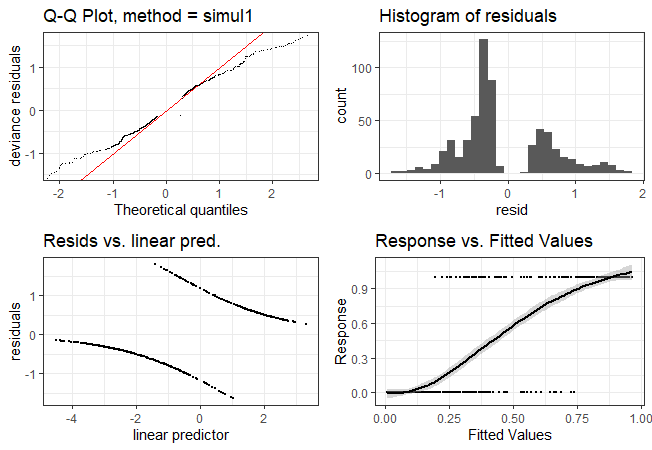


**Additional file 1: Figure S12:** Diagnostic plots for model fits of inpatient admission using the household dataset


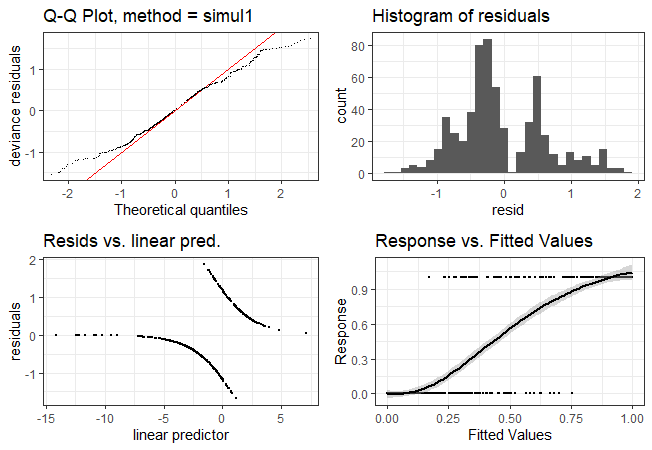


**Additional file 1: Figure S13:** Diagnostic plots for model fits of inpatient admission using the combined dataset
